# Whole Body Vibration Improves Spatial Memory, Anxiety-Like Behavior, and Motor Performance in Aged Male and Female Rats

**DOI:** 10.3389/fnagi.2021.801828

**Published:** 2022-01-21

**Authors:** Tamás Oroszi, Eva Geerts, Sietse F. de Boer, Regien G. Schoemaker, Eddy A. van der Zee, Csaba Nyakas

**Affiliations:** ^1^Department of Neurobiology, Groningen Institute for Evolutionary Life Sciences, University of Groningen, Groningen, Netherlands; ^2^Research Center for Molecular Exercise Science, University of Physical Education, Budapest, Hungary; ^3^Department of Cardiology, University Medical Center Groningen, Groningen, Netherlands; ^4^Behavioral Physiology Research Laboratory, Health Science Faculty, Semmelweis University, Budapest, Hungary

**Keywords:** passive physical exercise, object recognition, open field activity, muscle strength, healthy aging

## Abstract

Aging is a progressive process leading to functional decline in many domains. Recent studies have shown that physical exercise (PE) has a positive influence on the progression of age-related functional decline, including motor and brain functions. Whole body vibration (WBV) is a form of passive stimulation by mechanical vibration platforms, which offers an alternative for PE interventions, especially for aged individuals. WBV has been demonstrated to mimic the beneficial effects of PE on the musculoskeletal system, as well on the central nervous system. However, preclinical data with aged rodents are very limited. Hence, the purpose of this experiment was to investigate the effects of a 5-week WBV intervention with an aged animal model on memory functions, anxiety-related behavior, and motor performance. The 18-month old male (*N* = 14) and female (*N* = 14) Wistar rats were divided into two groups, namely, vibration and pseudo-vibration. Animals underwent a 5-week WBV intervention protocol with low intensity (frequency of 30 Hz and amplitude of 50–200 μm) stimulation. After 5 weeks, the following cognitive and motor tests were administered: open-field, novel and spatial object recognition, grip-hanging, and balance-beam. WBV-treated rats showed a decrease in their anxiety level in the open field test compared with those in the pseudo-treated controls. In addition, WBV-treated male animals showed significantly increased rearing in the open-field test compared to their pseudo controls. Spatial memory was significantly improved by WBV treatment, whereas WBV had no effect on object memory. Regarding motor performance, both grip strength and motor coordination were improved by WBV treatment. Our results indicate that WBV seems to have comparable beneficial effects on age-related emotional, cognitive, and motor decline as what has been reported for active PE. No striking differences were found between the sexes. As such, these findings further support the idea that WBV could be considered as a useful alternative for PE in case active PE cannot be performed due to physical or mental issues.

## Introduction

Aging is accompanied by an increased risk for various pathological conditions in the central nervous system such as reduced total brain volume (Salat et al., 1999) and blood flow velocity (Moreno-Torres et al., 2005), increased neurodegeneration (Sastry and Rao, 2000; Walhovd et al., 2011), or mitochondrial dysfunctions (Trifunovic and Larsson, 2008). Thereby, the aging process causes a progressive decline in cognitive functions. For instance, aging in rodents is associated with impaired learning and memory functions in behavioral tasks such as the Morris water maze (MWM) (Gallagher et al., 2015; Gocmez et al., 2016) and novel objects recognition (NOR) tasks (Arias-Cavieres et al., 2017; Zhang et al., 2017). In addition, the higher prevalence of anxiety and depression and/or hyperemotionality-like behavioral patterns in aged rodents have been observed in the open field (Mikael et al., 2007), the elevated-T maze (Aysegul et al., 2008), and the elevated-plus maze tasks (Pawel and Jolanta, 2002). Age-related decline in motor coordination and muscle strength is associated with the atrophy of muscle tissue, dysfunctions of muscle motor units due to failure at synaptic transmission, and hormonal changes and/or the combination of these. At present, several extensive reviews have summarized the most prevalent changes in aged rodents (Larsson et al., 2001; Ewa et al., 2008; Sam et al., 2014; Cho et al., 2016).

Physical activity is widely acknowledged as one of the most relevant and accessible low-cost treatments to promote general health benefits, support brain functions, and reduce the risk of neurodegenerative and other diseases associated with aging (Cotman and Engesser-Cesar, 2002; van Praag, 2005; Kramer and Erickson, 2007). In addition, results from animal studies suggest that exercise interventions have a positive influence on different types of memory functions, as well as emotional and motor aspects of behavior (Binder et al., 2004; Fulk et al., 2004; van Praag, 2005; Uysal et al., 2018). However, there is a growing interest in investigating new domains related to lifestyles and alternative exercise interventions to support and improve cognitive and motor capabilities of populations who are not motivated and/or unable to perform sufficient active exercise training. Whole body vibration (WBV) may offer a moderate and regular alternative type of passive exercise stimulation by mechanical vibration platforms. Many studies related to vibration have been published during the last decades and have shown beneficial and health-related effects of WBV on different domains. Physical fitness, especially in the musculoskeletal system, such as increased bone density (Slatkovska et al., 2010) and muscle strength (Annino et al., 2007; Annino et al., 2017), and increased hormonal responses have been reported (Bosco et al., 2000). Additionally, WBV has been considered as an alternative type of exercise for the elderly population to improve general fitness, mobility, and balance (Runge et al., 2000; Zhang et al., 2014).

Recent studies have shown that vibration may beneficially stimulate the cerebral cortical regions, as well other regions such as the hippocampus or prefrontal cortex, through the activation of muscle, tendon, and skin receptors and transmission of the vibration stimuli *via* the Goll and Burdach paths of the spinal cord (Hagbarth and Eklund, 1968; Bosco et al., 1999). Preclinical studies with rodents have found that WBV may stimulate the release of various neurotransmitters such as acetylcholine (Heesterbeek et al., 2017), serotonin (Ariizumi and Okada, 1985), and dopamine (Zhao et al., 2014) in different brain regions. Under pathological conditions in rodent models like after cerebral ischemia and stroke, WBV may also promote the expression of different neurotrophic factors and neurogenesis-related markers such as brain-derived neurotrophic factor (BDNF), insulin-like growth factor (IGF1), and doublecortin (DCX) (Huang et al., 2018; Raval et al., 2018). Also, vibration seems to be effective to alleviate depression-like symptoms in open field and elevated plus maze tests, degeneration of neurons, and pathological changes in microglia cells induced by the chronic restraint test (Peng et al., 2021). In addition, increased memory and motor functions in rodents have been also reported after 5 weeks of vibration treatments (Keijser et al., 2017; Boerema et al., 2018). These observations were taken in (young) adult rodents. In addition, a study in 12-month old female rats has shown that WBV reduces brain damage induced by the chronic stroke model and stimulates the release of BDNF (Raval et al., 2018). However, the neurobiological data of aged rodents are very limited, although WBV is supposed to be an important alternative for (top) aged individuals. To gain more insight into the impact of WBV on aged rodents, we investigated whether WBV can influence spatial memory, anxiety-like behavior, and motor performance in aged rats. Moreover, little attention has been given to sex differences in WBV studies. To address this, we designed a 5-week-long vibration intervention with low-intensity vibration exposure (frequency of 30 Hz and amplitude of 50–200 μm) in 18- to 20-month-old Wistar rats. After 5 weeks, a test battery was performed, including open field, novel, and spatial object recognition (NOR and SOR), balance beam, and grip hanging tests, and served as primary outcome measures for the behavior and motor performance of an animal. The age of 18–20 months was chosen because this age in rats comes with an early, but detectable, age-related deficits in learning, memory, and motor performance. Both male and female rats were used to reveal whether the efficiency of vibration treatment in age-related deficits depends on sex.

## Materials and Methods

### Animals

Twenty-eight Wistar rats (14 males and 14 females) were used in this experiment from our own breeding colony. Age of these animals was 18 months at the start of the experiment. Both male and female rats were randomly divided over two treated groups (male—WBV and female—WBV) and to two control groups (male—pseudo WBV and female—pseudo WBV), all *n* = 7 per group. Pseudo WBV rats were subjected to the same environmental exposure, including replacement and motor sound of the vibrating plate, but were not subjected to vibration. Animals were grouped together (i.e., two per cage) in their own home cages to avoid discomfort caused by social isolation. Food and water were available *ad libitum*. Animals were housed under normal laboratory conditions in 12–12 dark/light cycles (light on at 7:00 a.m.) with standard temperature (22 ± 2°C) and humidity (50 ± 10%). All procedures of this experiment were approved by the animal ethical committee of the University of Physical Education, and all animals were used in accordance with guidelines of the European Union Council Directive (86/609/European Economic Community). The health status of the animals was checked daily by researchers, and their bodyweight was monitored weekly during the experimental period.

### Procedure of Whole Body Vibration

Animals underwent a single vibration session of 10 min, five times per week during five consecutive weeks using a vibration platform (MarodyneLiV, Low Intensity Vibration; BTT Health GmbH; Germany) that offers constant frequency of 30 Hz and amplitude of 50–200 μm (vertical) for an object weighted only in the range of 20–120 kg. Therefore, it was essential to optimize the vibration platform for the rats by placing a metal dumbbell (25 kg) on the top of the vibration platform. During the training sessions, animals were placed into an empty cage [identical to the housing cage (36 × 18 × 23 cm), without woodchips] on the top of the vibration platform (i.e., directly on the top of the dumbbell). In addition, there were no social interactions between the animals of the different cages during the WBV sessions (i.e., the same cage mates were always treated together). The parameters of vibration with this experimental setting have been tested and verified by additional measurements (i.e., in the center and in the corners of the cage) using the accelerometer (frequency: 29.6 Hz; amplitude: 0.01–0.03 mm). Furthermore, animals did not receive prior habituation to the treatment procedure. It is important to note that animals were not constraint during the treatments, and they showed slightly excited behavior (i.e., unprompted motor activity such as walking or rearing) during the first 3–5 days of intervention. However, from the second week onward, animals did not show additional activity and remained in sitting or mainly in lying position in the cage without high degree of unprompted activity. All WBV sessions were conducted in a separated experimental room (i.e., under light conditions with same humidity and temperature parameters as the housing room) in the morning between 10 and 11 a.m. Animals did not have acute, short-term or long-term side effects by vibration. We adhered to the new reporting guidelines for WBV studies in animals (van Heuvelen et al., 2021).

### Test Procedures

**FIGURE 1:**
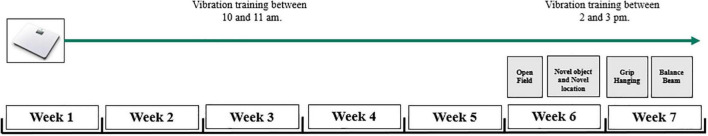
Experimental design. The 18-month-old male and female rats underwent 5 weeks of whole body vibration intervention with a single daily session of 10 min exposure, five times per week. After 5 weeks, a test battery was performed to assess anxiety-like behavior, memory functions, and motor performance. The used MarodyneLiv plate is depicted in the upper left corner.

After five consecutive weeks of vibration intervention, a 2-week-long test series, including novel and SOR, open field, grip strength, and balance beam test was conducted to assess memory functions, anxiety, and depression-related behavioral patterns, motor coordination, and muscle strength (Figure 1). Animals received the vibration exposure during the testing phase; however, all tests were always conducted 24 h following the last vibration session to avoid a direct effect of WBV on their performance. All tests were performed under dimmed light conditions in a quite test room between 10 a.m. and 2 p.m. During the weeks of the test procedures, WBV was given between 2 and 3 p.m.

### Open Field

Open field was performed to assess behavioral patterns induced by a novel environment. The test box was a circular area with a diameter of 80 cm, which was divided into 20 sectors by black circular and radial lines, and surrounded by a 45 cm tall wall. Rats were placed in the center of the arena at the start of the experiment and were exposed to the unfamiliar environment for 5 min. Video recordings were taken. The novel environment induced the vertical locomotor activity (i.e., rearing). Horizontal mobility was assessed by visual observation of video records by use of ELINE software (Nyakas et al., 1991). The test arena was cleaned out with 70% ethanol solution and dry paper tissue between all sessions. The following parameters were determined: frequency of line crossing (i.e., walking), the time spent between different sectors of the test arena (i.e., center time and wall time), and total number of rearings (i.e., they rose on their hind limbs supported by the wall or without the wall).

### Novel and Spatial Object Recognition

**FIGURE 2:**
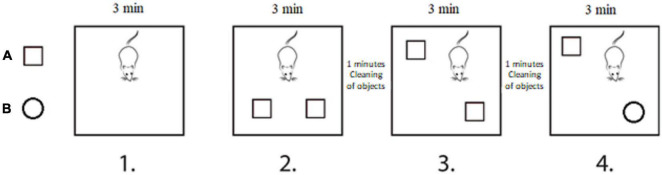
Novel and spatial object recognition test battery was performed to assess spatial and object memory. The test series consisted of four separated phases. Phase 1: rat was placed into the empty test box for 3 min to get familiarized to the test environment. Phase 2: two identical objects were placed into the test box in parallel position, and the rat was familiarized to the objects for 3 min. Phase 3: spatial object recognition: after cleaning, the two same objects were placed back in diagonal position, and the rat was allowed to explore the objects for 3 min. Phase 4: novel object recognition: after cleaning, objects were placed back in the diagonal position; however, one of the familiar objects was replaced by a novel object, and the rat was allowed to explore the objects for 3 min.

Novel and SOR test series were performed to analyze spatial and object memory described earlier by Ennaceur and Meliani (1992). In brief, this test battery consisted of four separated phases (Figure 2), each lasted for 3 min, with a 1 min break between phases 2 and 3, as well as between phases 3 and 4. In the first phase, the rat was placed into the empty test box and exposed to explore the test environment. In the second phase, the rat was familiarized to the test objects by placing two identical objects in the test box in parallel position. The same objects were placed back in the third phase (SOR); however the position of one of them was changed (i.e., same objects were placed back in the diagonal position). Finally, in the fourth phase (NOR), one of the familiar objects was replaced by a novel object. The roles of two sets of objects were randomly rotated through the procedures. Between all phases, the objects were removed from the test box and placed back after cleaning using 70% ethanol and dry paper. The animals who did not explore the objects (8 in total) were excluded at the final statistical analysis (SOR: 1 from the vibration/male group; NOR: 1 from vibration/female group and 1 from the pseudo vibration/female group, 3 from pseudo vibration/male group, 2 from the vibration/male group). Video recordings were taken during the entire procedure. For the familiarization phase to the objects, as well for the SOR and NOR test phases, exploration times of the objects were measured by visual observation of the video recordings using ELINE software. Preference time (recognition index in %) was calculated and served as the final outcome variable. Preference time was calculated by the following formula:


$$\frac{\mathrm{Time}\mathrm{spent}\mathrm{at}\mathrm{the}\mathrm{novel}\mathrm{object}\mathrm{or}\mathrm{relocated}\mathrm{object}}{\mathrm{Total}\mathrm{object}\mathrm{exploration}\mathrm{time}}\times 100.$$

In addition, total object exploration time, as well as active (time spent with locomotor activity such as walking and rearing in the test environment) and passive time (time spent with lying or sitting position or with grooming), was also determined to quantify unprompted motor activity during the novel and SOR test series.

### Balance Beam

The balance beam test (Song et al., 2006) was conducted three times for two consecutive days (1 day familiarization and 1 test day) to assess motor coordination. A 150 cm long and 4 cm wide wood beam was fixed 1 m above the floor in a horizontal position. The home cage of the animals was placed at the end of the beam serving as motivation and target for the animals.

Rats were familiarized to the task on the first day by two consecutive progressive trials and one full test trial (50, 100, and 150 cm walking distance). On the second day, rats underwent two familiarization trials (50 and 100 cm) and three complete trials (150 cm). Mean of these three complete trials of the second day was used as the final outcome measure. One animal was unable or/and unwilling to walk over the beam and was excluded from the final analysis (1 from the pseudo vibration/male group). The balance beam setup was cleaned by 70% ethanol solution and dry paper towel after each test.

### Grip Hanging Test

Similar to the balance beam test, the grip hanging test was also performed two times on two consecutive days (3 trials per day) to assess the muscle strength endurance of the forelimbs (Shear et al., 1998). The first day served as the familiarization day. Animals were picked up and supported by their trunk and allowed to grasp a suspended steel wire (2 mm diameter, 35 cm long, and 50 cm above the surface) by their forepaws, and the time until falling was recorded manually by a second researcher. Animals were rotated every day through the three trials to ensure enough recovery time between two trials and avoid potential injuries caused by muscle fatigue. After each trial, the test apparatus was cleaned by 70% ethanol and dry paper tissue. The mean of the three best trials from the first and/or second days were used as final outcome measure. One animal was unable or/and unwilling to learn the test procedure and was excluded from the final analysis (1 from the pseudo vibration/male group).

### Statistical Analyses

The statistical analysis was performed using Statistica 13.2 software. Factorial ANOVA (factor 1: intervention: vibration/pseudo vibration; factor 2: sex: male/female) followed by the Tukey’s *post hoc* test was used to reveal statistical differences between the experimental groups in case of all outcome variables.

In addition, in case of the NOR and SOR tests, preference time was compared with the chance level of 50% by an independent sample *t*-test. This comparison was added to determine whether a group mastered the test.

For revealing the interaction between sensory and motor domains in the motor tests vs. cognitive and behavioral tests, a parametric correlation analysis was used. Statistical significance was set at *p* < 0.05. Graphs were made by using GraphPad Prism 8 software. Data are expressed as mean ± SEM.

## Results

### Open Field

**FIGURE 3:**
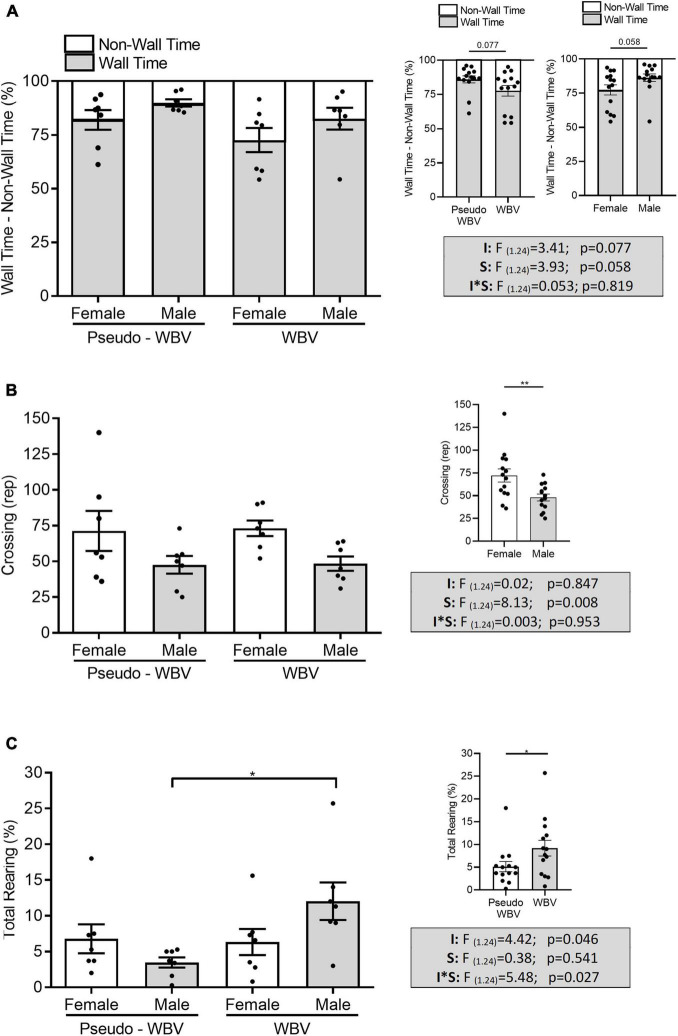
The effects of whole body vibration on exploratory and anxiety-related behavior in the open field test in aged male and female rats. Data are presented as percentage of wall vs. non-wall time (i.e., they are reciprocal of each other). All animals spent more than 50% of the total time in the wall zone. Vibration-treated animals showed a strong tendency (*p* = 0.077) of decreased time spent in the wall zone and increased time spent in the center zone **(A)**. The number of crossings significantly increased in the female animals **(B)**. Time spent with rearing increased significantly in the vibration-treated male group compared with the pseudo-treated male group **(C)**. Two-way ANOVA was used for analyzing these results. Pseudo-whole body vibration (*n* = 14, 7 females and 7 males); whole body vibration (*n* = 14, 7 females and 7 males). F, F statistical value; I, main effect of intervention (vibration/pseudo vibration); S, main effect of sex (male/female); I*S, interaction effect of intervention and sex. Data are depicted as mean ± SEM. *p < 0.05 and **p < 0.01.

The open field test was performed to assess exploratory and anxiety-related behavior. Two-way factorial ANOVA revealed a strong tendency of increased non-wall time/decreased wall time in the vibration-treated groups compared with that in the pseudo vibration control groups (*F*_(1_._24)_ = 3.41; *p* = 0.077) (Figure 3A). Significant increase of rearing activity was observed by vibration intervention (*F*_(1_._24)_ = 4.428; *p* = 0.046). In addition; a significant increase of the interaction effect of intervention and sex on the rearing activity was also found (*F*_(1_._24)_ = 5.48; *p* = 0.027); vibrated male rats showed a significantly higher level of vertical activity compared with male pseudo vibration rats (*post hoc p* = 0.021) (Figure 3C). Vertical activity was not affected in the female animal by vibration treatment.

In addition, a tendency of increased non-wall time/decreased wall time was observed in the female animals compared with male animals (*F*_(1_._24)_ = 3.93; *p* = 0.058). Furthermore, significant higher number of crossings was also detected in the female animals compared to the male animals (*F*_(1_._24)_ = 8.13; *p* = 0.008) (Figure 3B).

### Spatial Object Recognition and Novel Location Test

**FIGURE 4:**
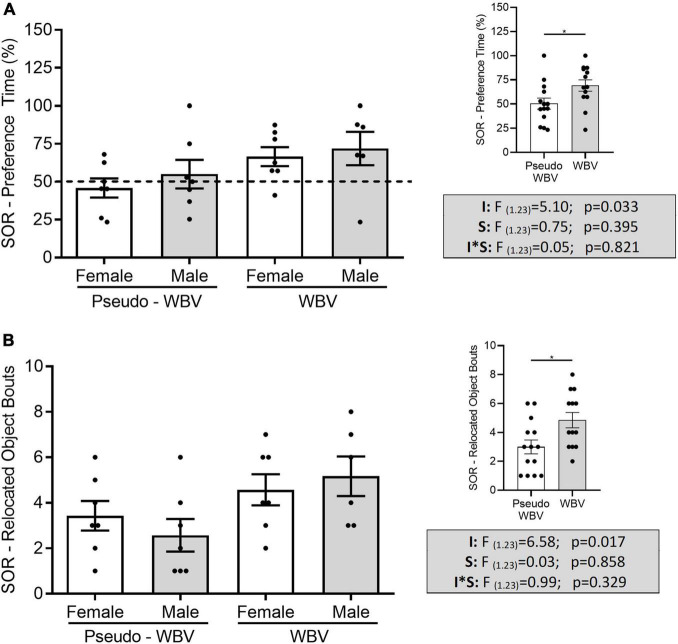
Effects of whole body vibration on spatial memory in the spatial object relocation test. Preference time **(A)** and relocated object bouts **(B)** were significantly increased in vibration treated groups compared with control groups. Two-way ANOVA was used for analyzing these results. Pseudo whole body vibration (*n* = 14, 7 females and 7 males) and whole body vibration (*n* = 13, 7 females and 6 males). Dotted line: reference line of 50% for random exploration. F, F statistical value; I, main effect of intervention (vibration/pseudo vibration); S, main effect of sex (male/female); I*S, interaction effect of intervention and sex. Data are depicted as mean ± SEM. *p < 0.05.

Spatial and novel object recognition tests were performed to assess the spatial and object recognition memory of the rats. During the SOR task, two-way ANOVAs showed a significant increase of preference time in the vibration-treated groups (*F*_(1_._23)_ = 5.103; *p* = 0.033) (Figure 4A). In addition, vibration also significantly influenced the ability in the SOR test to discriminate between the familiar and novel locations of the objects relative to chance level (male *p* = 0.007; female *p* = 0.038) (*p < 0.05, *t*-test for related samples, i.e., it reflects significant difference compared with the chance level of 50%). This discrimination parameter was not affected in the pseudo vibration groups (male *p* = 0.536; female *p* = 0.615). Similarly, a significant increase in the frequency of exploration of the relocated object (i.e., how many times the animals touched or smelt the object?) was also detected in the vibration-treated groups (*F*_(1_._23)_ = 6.586; *p* = 0.017) (Figure 4B). This parameter was not altered in case of familiar object.

**FIGURE 5:**
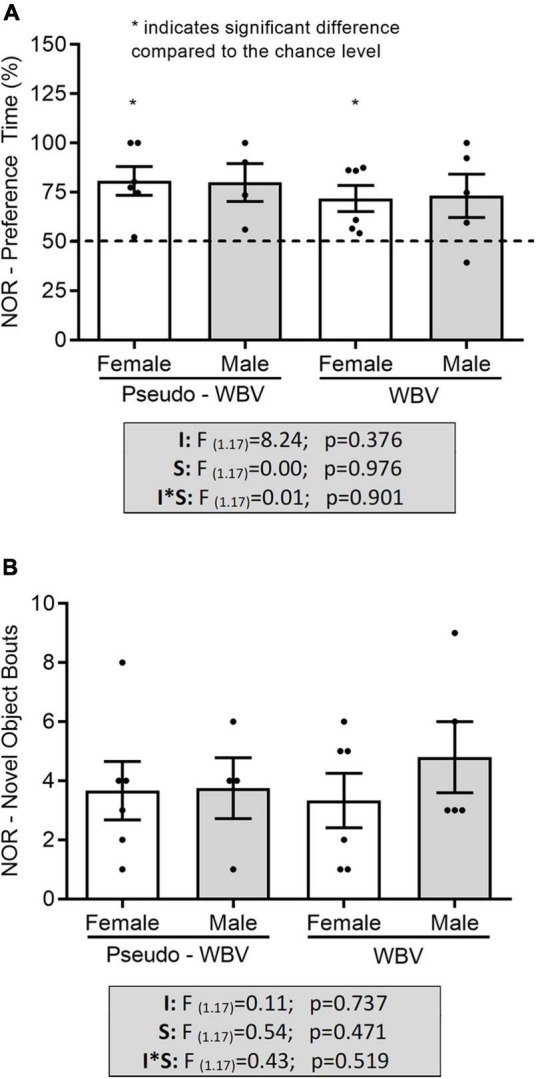
Effects of whole body vibration on object memory in the novel object recognition test. Preference time and number of novel object bouts had no significant difference among all groups **(A,B)**. Preference time showed significant difference in the female animals compared with the 50% of chance level **(A)**. Two-way ANOVA was used for the analysis of group interactions, and the independent sample *t*-test was used for comparison to the chance level. Pseudo whole body vibration (*n* = 10, 6 females and 4 males) and whole body vibration (*n* = 11, 6 females and 5 males). Dotted line: reference line of 50% for random exploration. F, F statistical value; I, main effect of intervention (vibration/pseudo vibration); S, main effect of sex (male/female); I*S, interaction effect of intervention and sex. Data are depicted as mean ± SEM. *p < 0.05.

No difference in the preference time and in the number of objects bouts during the NOR task was found between all groups (Figures 5A,B). However, the discrimination pattern relative to the 50% of chance level was significantly increased in the female animals (pseudo vibration *p* = 0.008; vibration *p* = 0.021) (*p < 0.05, *t*-test for related samples, i.e., it reflects significant difference compared with the chance level of 50% – *depicted on Figure 5A). Animals did not show increased preference time and number of bouts for the objects during the familiarization phase (i.e., familiarization phase before the SOR and NOR phases when rats were subjected to two same objects) (data are not shown).

In addition, object exploration time (i.e., time spent at both objects), active (time spent with unprompted motor activity such as walking or rearing), and passive time (time spent with sitting or lying or grooming) were also determined during the test phases (see Table 1). A significant effect of interaction of intervention and sex was observed in active time during the SOR phase (*F*_(1.23)_ = 4.97; *p* = 0.036). Vibration-treated male rats showed significantly more active time compared with the pseudo-treated male rats. Main effects of intervention and sex in the object exploration time, active, and passive time were not significantly altered.

**TABLE 1:** Effects of whole body vibration on object exploration time (time spent at both familiar object and novel/replaced object), on active time (i.e., unprompted motor activity such as walking or rearing), and on passive time (i.e., sitting and lying and time spent with grooming) during the spatial and novel object recognition tests.

		Pseudo - WBV		WBV	
		Female	Male	Female	Male
Spatial object recognition	Object Exploration Time	8.92 ± 2.11	9.64 ± 3.19	9.07 ± 1.65	11.51 ± 2.69
	Active Time	42.65 ± 5.04	29.28 ± 6.54*	39.70 ± 4.73	49.06 ± 2.73*
	Passive Time	49.78 ± 6.41	61.31 ± 9.08	50.27 ± 6.56	40.18 ± 3.24
Novel object recognition	Object Exploration Time	8.93 ± 2.23	13.10 ± 3.72	14.61 ± 3.06	12.86 ± 4.79
	Active Time	31.52 ± 11.21	31.52 ± 15.77	38.13 ± 4.99	37.60 ± 4.04
	Passive Time	56.20 ± 12.00	55.15 ± 18.95	47.25 ± 6.35	50.10 ± 7.32

*A significant effect of interaction of intervention, and sex was observed in active time during the spatial object recognition test. Vibration-treated male animals showed significantly higher active time compared with the pseudo-treated animals. Other parameters were not significantly altered. Data are represented in percentage of total test duration. Spatial object recognition/novel object recognition: Pseudo whole body vibration (n = 14/10, 7/6 females and 7/4 males) and whole body vibration (n = 13/11, 7/6 females and 6/5 males). Data are depicted as mean ± SEM.*

*Asterisk indicates p < 0.05; significant difference between the marked groups.*

### Grip Strength and Balance Beam

**FIGURE 6:**
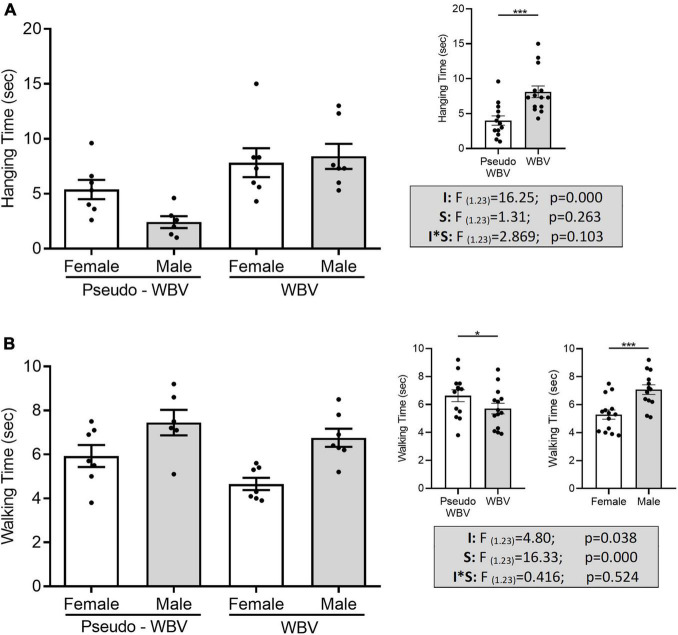
Effects of whole body vibration on motor performance in the grip hanging **(A)** and balance beam **(B)** tests. Hanging time was significantly increased in vibration-treated animals compared to the pseudo vibration-treated animals **(A)**. Walking time on the beam **(B)** was significantly decreased in vibration-treated animals compared with the pseudo vibration-treated animals, as well in the female animals compared with the male animals. Grip hanging/balance beam: pseudo whole body vibration (*n* = 13/13, 7/7 females and 6/6 males) and whole body vibration (*n* = 14/14, 7/7 females and 7/7 males). F, F statistical value; I, main effect of intervention (vibration/pseudo vibration); S, main effect of sex (male/female); I*S, interaction effect of intervention and sex. Data are depicted as mean ± SEM. *p < 0.05 and ***p < 0.001.

Grip hanging and balance beam tests were conducted for the assessment of motor performance and served as outcome measurements of motor coordination and grip strength. Two-way ANOVA revealed that hanging time was significantly affected by vibration treatment (*F*_(1_._23)_ = 16.25; *p* = 0.000) (Figure 6A). A significant increase in balance beam performance was found in the vibration-treated groups compared with pseudo control groups (*F*_(1_._23)_ = 4.80; *p* = 0.038), as well in female animals compared with the male animals (*F*_(1_._23)_ = 16.33; *p* = 0.000) (Figure 6B).

The assessment the interaction between the motor parameters in these motor tests vs. anxiety-like behavior in the open field test was determined by the correlation analysis. It was found that muscle strength positively correlated with the anxiety-like behavior in the open field test suggesting the development of a positive impact of motor abilities on anxiety (hanging time vs. open field non-wall time: *r* = 0.408; *p* = 0.034). In addition, a same strong tendency of correlation (walking time vs. open field non-wall time: *r* = −0.328; *p* = 0.094) was detected between the motor coordination in the balance beam test and anxiety-like behavior in the open field test. In addition, body weight of the animals was not significantly changed during the intervention.

## Discussion

This experiment investigated the impact of long-term (5 weeks) vibration training with low intensity and of a sinusoidal nature on cognitive parameters, anxiety-like behavior, and motor performance. The results of this study showed that WBV improved anxiety-like behavior in the open field test, spatial memory in the spatial object relocation test, and motor performance in grip hanging and balance beam tests. Taken together, our data demonstrate that a daily 10 min vibration protocol for 5 weeks using the MarodyneLiv vibration platform is able to improve brain functioning in aged rats.

Our results in the context of improved motor performance are in line with previous findings using the MarodyneLiV plate. Human studies using this vibration platform with low amplitude show that it can improve muscular and skeletal functions (Petryk et al., 2017). In addition, preclinical studies with rodents have been reported that interventions using the low-magnitude approach with the Marodyne platform improve neuromuscular dynamics and muscle strength (Mettlach et al., 2014), alleviate adipose tissue dysfunctions, enhance glucose metabolism (Patel et al., 2017), and accelerate muscle healing (Corbiere et al., 2017). In general, low-intensity vibration has been demonstrated to improve muscular functions both in humans (Gilsanz et al., 2006; Muir et al., 2011) and in animals (Xie et al., 2008; McKeehen et al., 2013) and has been considered as a low-cost treatment and preventive alternative for active physical exercise (PE).

The open field test is one of the most popular tests for studying anxiety-like and exploratory behavior of the rodent, and it is based on measuring natural approach/avoidance behavior in a novel environment. Our findings in the open field test revealed that vibration-treated animals spent less time in the outer wall zone of the arena (and spent more time in the center part of the arena) compared with their pseudo counterparts. Also, vibration-treated male animals showed significant higher vertical activation compared with the male pseudo-treated group. In general, these findings indicate that WBV may have beneficial effects on anxiety and motor decline accompanied by aging. Additional studies using more specific anxiety tests are needed to confirm our interpretation of the open field data. However, it is known that aging increases anxiety-related indices (i.e., decreased locomotion and rearing, increased immobility time, more spent time in the outer zone) and decreases antianxiety indices in the open field test (i.e., less spent time in the inner zone) (Vasquez et al., 1983; Dorce and Palermo-Neto, 1994; Boguszewski and Zagrodzka, 2002; Amirazodi et al., 2020). In addition, indices in the open field test such as unprompted physical activity (i.e., horizontal ambulation and rearing) requires higher level of muscle work and coordination of hind limbs; thereby, these outcomes represent motor performance in explorative tasks and their gradual decline from the age of 16 months onward has been already reported (Mikael et al., 2007) in the open field task.

In addition, our data also revealed that the number of crossings (i.e., walking distance) was significantly affected by sex. Female animals covered more distance in the open field arena compared with the male animals. In addition, a similar tendency was also detected in the wall vs. non-wall time; the female animals spent more time in the center of the arena (and less time at the wall of the area) compared with their male counterparts. Similar gender differences in open field behavior have been already reported by others (Johnston and File, 1991; Domonkos et al., 2017; Scholl et al., 2019).

To the best of our knowledge, there is only one study regarding the effects of WBV on anxiety and exploratory-like behaviors in the open field test, in which, in accordance to our own results, vibration with the frequency of 30 Hz and amplitude of 4.5 mm has been reported to improve depression-like behavior in rats induced by the restrain stress model (Peng et al., 2021). Compared with our own protocol (frequency of 30 Hz and amplitude of 200–250 μm), this observation indicates that frequency may play a more crucial role to obtain beneficial effects on the level of the behavior of the rodent than amplitude.

Ample studies have shown that aged rats have learning and memory impairments (Gallagher et al., 2015; Gocmez et al., 2016; Arias-Cavieres et al., 2017; Zhang et al., 2017). SOR performance, which reflects the hippocampus-dependent spatial component of memory, shows a decline from the age of 18 months onward in rats. The SOR test in our study revealed that vibration-treated animals were able to distinguish the position of the replaced object in contrast to the pseudo-treated animals. Hence, WBV seems to be able to improve this type of memory in aged rats. Also, vibration-treated male animals showed significantly higher unprompted motor activity during the SOR phase compared with the pseudo-treated male animals. This is in line with the known combined effect of WBV on brain and motor functions (Oroszi et al., 2020 and references therein). In contrast to SOR, the NOR test was not affected by WBV, although this finding was based on rather low numbers of animals, which showed sufficient exploratory behavior (as mentioned in section “Materials and Methods”). Novel object memory, which largely depends on the perirhinal cortex (Aggleton et al., 2010), was not affected in old rats (Cavoy and Delacour, 1993; van der Staay, 2002). Similar results have been described in mice. Both 12- and 24-month old mice have a decline of object relocation memory, but object memory remained intact at both age (Wimmer et al., 2012; Li et al., 2015). Overall, it seems that object location memory (spatial memory) in rodents is more sensitive to the aging process than the ability to recognize and remember the features of an object. Our findings are in line with this observation. WBV has been shown to positively affect the hippocampus (Cariati et al., 2021; Peng et al., 2021), which is also in line with our findings on spatial memory. One article reported that WBV with 5 min sessions (but not with 30 min sessions) enhanced the performance in the NOR tests in young mice (3 months of age), whereas the SOR test was not affected (Keijser et al., 2017). The difference in effect of WBV on SOR or NOR could be strain or age specific (young vs. old) but also depends on the applied WBV protocol.

Our findings suggest that vibration could be an alternative for active exercise to improve certain domains of memory function. Beneficial effects of aerobic exercise such as treadmill running have been reported on spatial memory and learning functions of aged rodents in various tasks such as MWM (van Praag, 2005; Kurucz et al., 2018), as well in spatial object location tests (Cechella et al., 2014; Snigdha et al., 2014). Vibration is typically considered as a form of low-intensity aerobic exercise and its effects on memory functions have been investigated in clinical studies where vibration seems to be beneficial to improve executive functions and other cognitive domains (Regterschot et al., 2014; den Heijer et al., 2015).

Both muscle strength and motor coordination were improved by vibration treatment, and these findings indicate that vibration prevents or reverses a decline of motor performance. It has been reported that the disturbance of balance beam performance in rats first appears to be visible at the age of 15–20 months, but it becomes evident at more advanced age (between 20 and 25 months) (Mikael et al., 2007). Beneficial effects of active exercise on balance beam performance have been observed at the age of 18 and 24 months in rats (Silveira et al., 2019). In addition, the preventive effects of exercise have been reported to eliminate and delay the progressive and generalized loss of muscle mass and functions through the life of Wistar rats (Hernández-Álvarez et al., 2019). For some decades, the motor component of vibration on the skeletal muscle has been broadly investigated, and it has been known as general evidence that vibration primarily stimulates the plasticity of the skeletal muscle and modulates its motor units *via* the activation of muscle and skin receptors (Hagbarth and Eklund, 1968; Bosco et al., 1999). Thereby, vibration represents a strong stimulation to increase muscle strength and coordination both in humans (Annino et al., 2007, 2017) and rodents (Mettlach et al., 2014; Corbiere et al., 2017; Keijser et al., 2017). In addition, WBV has been considered as an alternative type of exercise for elderly populations to improve muscle strength and coordination (Runge et al., 2000; Zhang et al., 2014). Hence, our results obtained in the balance beam and grip strength tests confirm the efficiency of WBV training in the context of neuromuscular plasticity and adaptation.

A significant correlation was found between hanging time in the grip test and open field test parameters potentially related to depression. The higher the muscle strength, the less time was spent at the wall zone of the open field arena (and more time at the center of area). Preclinical data are limited in this context; however, according to a recent review and meta-analysis in human patients (Zasadzka et al., 2021), the relationship between lowered hand grip strength and intensified depressive symptoms has reported as a relevant and important sign to detect and screen depression. The finding that WBV improves muscle strength and decreases anxiety-like behavior favors its use as an (additional) intervention to battle depression.

## Conclusion

In accordance with the literature, our findings indicate the effectiveness of long-term vibration training with low magnitude in improving anxiety-related indices, cognitive abilities like spatial memory, as well in enhancement of neuromuscular adaptation in 18-month-old rats. It adds to the growing literature of WBV being an intervention that improves motor and brain functioning (like active PE). These data suggest that WBV could be considered to replace or to be combined with active PE, notably in situations where active PE is difficult to perform. Also, WBV may have the potential to counteract the progression of age-related functional decline, even in diseases such as Alzheimer’s or Parkinson’s diseases. However, further (preclinical) experiments are still needed to determine and understand the interactions of different variables of WBV designs referred as “Big Five”: the vibration amplitude, vibration frequency, method of application, session/duration frequency, and the total intervention duration (Oroszi et al., 2020). In addition, it will be important to reveal and understand the underlying molecular and cellular processes related to muscle, as well to brain functioning.

## Data Availability Statement

The original contributions presented in the study are included in the article/Supplementary Material, further inquiries can be directed to the corresponding author.

## Ethics Statement

The animal study was reviewed and approved by the Animal Ethical Committee of University of Physical Education.

## Author Contributions

EZ, SB, RS, and CN contributed to the conception and design of the study. TO performed the animal experiment. TO and EG analyzed and organized the data, did the statistical analysis, and wrote the first draft of the manuscript. All authors contributed to the final version of manuscript and approved the final version.

## Conflict of Interest

The authors declare that the research was conducted in the absence of any commercial or financial relationships that could be construed as a potential conflict of interest.

## Publisher’s Note

All claims expressed in this article are solely those of the authors and do not necessarily represent those of their affiliated organizations, or those of the publisher, the editors and the reviewers. Any product that may be evaluated in this article, or claim that may be made by its manufacturer, is not guaranteed or endorsed by the publisher.
