# Supplementary material for: Whole Body Vibration Improves Spatial Memory, Anxiety-Like Behavior, and Motor Performance in Aged Male and Female Rats
Source: Front Aging Neurosci. 2022 Jan 21;13:801828. doi: 10.3389/fnagi.2021.801828 (PMC8815031; doi:10.3389/fnagi.2021.801828)
Supplement: Supplementary file 1 [file Data_Sheet_1.pdf]

Table 1. Descriptive Statistics

Variable		N	Mean	Median	Min	Max	Std.Dev	SEM
OF Wall Time	All	28	81.81	86.45	54.3	96	12.87	2.43
	pWBV♂	7	89.92	88.5	85.5	96	4.29	1.62
	pWBV♀	7	82.02	86.8	61.3	93.7	12.14	4.59
	WBV♂	7	82.64	86.4	54.4	95.1	13.46	5.08
	WBV ♀	7	72.65	79.5	54.3	91.5	14.91	5.63
OF Non Wall Time	All	28	18.18	13.55	4	45.7	12.86	2.43
	pWBV♂	7	10.07	11.5	4	14.5	4.29	1.62
	pWBV♀	7	17.97	13.20	6.3	38.70	12.14	4.59
	WBV♂	7	17.35	13.60	4.9	45.6	13.46	5.08
	WBV ♀	7	27.34	20.50	8.5	45.7	14.91	5.63
OF Crossings	All	28	60.1	55.5	25	140	24.52	4.63
	pWBV♂	7	47.57	50	25	73	16.34	6.17
	pWBV♀	7	71.28	56	36	140	37	13.98
	WBV♂	7	48.42	45	31	64	13.17	4.98
	WBV ♀	7	73.14	73	52	91	14.46	5.46
OF Rearing Time	All	28	7.15	5.3	0.3	25.7	5.73	1.08
	pWBV♂	7	3.47	3.7	0.3	5.3	1.89	0.71
	pWBV♀	7	6.78	5.3	2	18	5.33	2.01
	WBV♂	7	12.02	11.3	3	25.7	6.95	2.62
	WBV ♀	7	6.32	6.6	0.8	15.6	4.82	1.82
Grip Hanging	All	27	6.14	6	1	15	3.46	0.66
	pWBV♂	6	2.41	2.3	1	4.6	1.3	0.53
	pWBV♀	7	5.38	5.3	2.6	9.6	2.32	0.87
	WBV♂	7	8.4	7.3	5.3	13	3.02	1.14
	WBV ♀	7	7.82	7.3	4.3	15	3.48	1.31
Balance Beam	All	27	6.15	6.2	3.8	9.2	1.51	0.29
	pWBV♂	6	7.45	7.35	5.1	9.2	1.42	0.58
	pWBV♀	7	5.92	5.7	3.8	7.5	1.31	0.49
	WBV♂	7	6.75	6.4	5.2	8.5	1.09	0.41
	WBV ♀	7	4.65	4.3	3.9	5.6	0.74	0.28
NOR Preference	All	21	76.26	77.48	39.28	100	18.43	4.02
	pWBV♂	4	79.93	81.87	56	100	19.32	9.66
	pWBV♀	6	80.77	78.88	52.21	100	17.90	7.31
	WBV♂	5	73.19	74.77	39.28	100	24.59	11.00
	WBV ♀	6	71.85	73.40	54.13	87.40	16.19	6.61
NOR Novel Bouts	All	21	3.85	4	1	9	2.26	0.49
	pWBV♂	4	3.75	4	1	6	2.06	1.03
	pWBV♀	6	3.66	3.5	1	8	2.42	0.98
	WBV♂	5	4.8	3	3	9	2.68	1.2
	WBV ♀	6	3.33	3.5	1	6	2.25	0.91
NOR Familiar Bouts	All	21	3.14	3	0	11	2.51	0.54
	pWBV♂	4	3	2.5	1	6	2.16	1.08
	pWBV♀	6	3.33	2.5	0	11	4.08	1.66
	WBV♂	5	2.8	3	0	5	1.92	0.86
	WBV ♀	6	3.33	3.5	1	5	1.63	0.66
NOR Object Exploration	All	21	12.28	11.30	1.70	29.90	7.62	1.6
	pWBV♂	4	13.10	13.10	5.00	21.20	7.44	3.72
	pWBV♀	6	8.93	9.20	1.70	15.10	5.46	2.23
	WBV♂	5	12.86	11.10	2.80	29.90	10.71	4.79
	WBV ♀	6	14.61	12.80	7.60	27.00	7.50	3.06
NOR Active Time	All	21	35.84	37.10	2.80	80.50	19.95	4.35
	pWBV♂	4	31.52	22.10	7.50	74.40	31.54	15.77

	**pWBV♀**	6	34.98	26.65	2.80	80.50	27.47	11.21
	**WBV♂**	5	37.60	37.50	24.30	49.40	9.05	4.04
	**WBV ♀**	6	38.13	38.35	16.40	52.80	12.23	4.99
**NOR Passive Time**	**All**	21	51.99	48.30	5.00	95.60	23.68	5.16
	**pWBV♂**	4	55.15	64.60	5.00	86.40	37.90	18.95
	**pWBV♀**	6	56.20	62.60	8.20	95.60	29.41	12.00
	**WBV♂**	5	50.10	48.30	29.40	70.50	16.37	7.32
	**WBV ♀**	6	47.25	42.55	33.90	75.80	15.56	6.35
**SOR Preference**	**All**	27	59.39	57.35	23.40	100	22.75	4.37
	**pWBV♂**	7	55.00	50	25.37	100	25.03	9.46
	**pWBV♀**	7	45.84	45.39	23.40	68	16.76	6.33
	**WBV♂**	6	71.93	76.77	23.43	100	26.94	11.00
	**WBV ♀**	7	66.57	62.5	41.05	87.37	16.63	6.28
**SOR NovelBouts**	**All**	27	3.88	4	1	8	2.04	0.39
	**pWBV♂**	7	2.57	2	1	6	1.90	0.71
	**pWBV♀**	7	3.42	3	1	6	1.71	0.64
	**WBV♂**	6	5.16	5	3	8	2.13	0.87
	**WBV ♀**	7	4.57	4	2	7	1.81	0.68
**SOR Familiar Bouts**	**All**	27	3.22	3	0	6	2.00	0.38
	**pWBV♂**	7	3	2	0	6	2.38	0.89
	**pWBV♀**	7	3.42	3	0	6	1.90	0.71
	**WBV♂**	6	2.66	3	0	5	1.96	0.80
	**WBV ♀**	7	3.71	4	0	6	2.05	0.77
**SOR – Total Object Exploration**	**All**	27	9.72	9.70	1.40	27.20	6.12	1.17
	**pWBV♂**	7	9.64	6.70	3.30	27.20	8.46	3.19
	**pWBV♀**	7	8.92	7.50	2.30	17.50	5.60	2.11
	**WBV♂**	6	11.51	10.00	4.30	23.90	6.58	2.69
	**WBV ♀**	7	9.07	10.30	1.40	13.60	4.37	1.65
**SOR Active Time**	**All**	27	39.84	40.90	15.00	67.20	14.39	2.76
	**pWBV♂**	7	29.28	21.10	15.00	54.40	17.31	6.54
	**pWBV♀**	7	42.65	40.30	29.20	67.20	13.35	5.04
	**WBV♂**	6	49.06	51.65	40.80	57.20	6.70	2.73
	**WBV ♀**	7	39.70	43.30	23.30	54.60	12.53	4.73
**SOR Passive Time**	**All**	27	50.76	53.10	18.30	81.70	18.37	3.53
	**pWBV♂**	7	61.31	73.90	18.30	81.70	24.02	9.08
	**pWBV♀**	7	49.78	56.90	19.70	64.40	16.98	6.41
	**WBV♂**	6	40.18	38.45	31.90	54.80	7.95	3.24
	**WBV ♀**	7	50.27	40.20	34.20	72.20	17.37	6.56

Table 2. Two Way Factorial ANOVA Outcomes

Variable	Interaction	F	p	Observed Power
**OF Wall Time**	Control/Vibration	F (1.24)= 3.41	0.077	0.425
	Male/Female	F (1.24)= 3.93	0.058	0.477
	Control/WVB/male/Female	F (1.24)= 0.053	0.819	0.055
**OF Non Wall Time**	Control/Vibration	F (1.24)= 3.41	0.077	0.425
	Male/Female	F (1.24)= 3.93	0.058	0.477
	Control/WVB/male/Female	F (1.24)= 0.053	0.819	0.055
**OF Wall Bouts**	Control/Vibration	F (1.24)= 0.091	0.764	0.059
	Male/Female	F (1.24)= 0.018	0.018	0.676
	Control/WVB/male/Female	F (1.24)= 0.498	0.487	0.104
**OF NonWall Bouts**	Control/Vibration	F (1.24)= 1.37	0.252	0.203
	Male/Female	F (1.24)= 13.94	0.001	0.947

	Control/WVB/male/Female	F (1.24)= 0.102	0.751	0.060
**OF Crossing**	Control/Vibration	F (1.24)= 0.025	0.874	0.052
	Male/Female	F (1.24)= 8.130	0.008	0.781
	Control/WVB/male/Female	F (1.24)= 0.003	0.953	0.050
**OF Rearing Time**	Control/Vibration	F (1.24)= 4.428	0.046	0.523
	Male/Female	F (1.24)= 0.384	0.541	0.091
	Control/WVB/male/Female	F (1.24)= 5.485	0.027	0.613
**Grip**	Control/Vibration	F (1.23)= 16.25	0.000	0.971
	Male/Female	F (1.23)= 1.316	0.263	0.195
	Control/WVB/male/Female	F (1.23)= 2.869	0.103	0.368
**Balance Beam**	Control/Vibration	F (1.23)= 4.805	0.038	0.555
	Male/Female	F (1.23)= 16.33	0.000	0.971
	Control/WVB/male/Female	F (1.23)= 0.416	0.524	0.094
**NOR Preference**	Control/Vibration	F (1.17)= 0.824	0.376	0.137
	Male/Female	F (1.17)= 0.0008	0.976	0.050
	Control/WVB/male/Female	F (1.17)= 0.015	0.901	0.051
**NOR Novel Bouts**	Control/Vibration	F (1.17)= 0.115	0.737	0.061
	Male/Female	F (1.17)= 0.541	0.471	0.106
	Control/WVB/male/Female	F (1.17)= 0.431	0.519	0.095
**NOR FamiliarBouts**	Control/Vibration	F (1.17)= 0.006	0.934	0.050
	Male/Female	F (1.17)= 0.129	0.722	0.063
	Control/WVB/male/Female	F (1.17)= 0.006	0.934	0.050
**NOR Object Exploration Time**	Control/Vibration	F (1.17)= 0.608	0.446	0.114
	Male/Female	F (1.17)= 0.119	0.734	0.062
	Control/WVB/male/Female	F (1.17)= 0.720	0.407	0.126
**NOR Total Exploration Time**	Control/Vibration	F (1.17)=0.422	0.524	0.094
	Male/Female	F (1.17)=0.004	0.944	0.050
	Control/WVB/male/Female	F (1.17)=0.017	0.895	0.051
**NOR Active Time**	Control/Vibration	F (1.17)= 0.235	0.633	0.074
	Male/Female	F (1.17)= 0.044	0.836	0.054
	Control/WVB/male/Female	F (1.17)= 0.023	0.879	0.052
**NOR Passive Time**	Control/Vibration	F (1.17)= 0.389	0.540	0.090
	Male/Female	F (1.17)= 0.006	0.936	0.050
	Control/WVB/male/Female	F (1.17)= 0.030	0.864	0.053
**SOR Preference**	Control/Vibration	F (1.23)= 5.103	0.033	0.580
	Male/Female	F (1.23)= 0.759	0.395	0.132
	Control/WVB/male/Female	F (1.23)= 0.051	0.821	0.055
**SOR Novel Bouts**	Control/Vibration	F (1.23)=6.586	0.017	0.690
	Male/Female	F (1.23)= 0.032	0.858	0.053
	Control/WVB/male/Female	F (1.23)= 0.994	0.329	0.159
**SOR Familiar Bouts**	Control/Vibration	F (1.23)= 0.0008	0.976	0.050
	Male/Female	F (1.23)= 0.838	0.369	0.141
	Control/WVB/male/Female	F (1.23)= 0.147	0.704	0.065
**SOR Exploration**	Control/Vibration	F (1.23)=0.165	0.687	0.067
	Male/Female	F (1.23)=0.406	0.530	0.093
	Control/WVB/male/Female	F (1.23)=0.121	0.730	0.062
**SOR Total Exploration Time**	Control/Vibration	F (1.23)=1.874	0.184	0.259
	Male/Female	F (1.23)=0.003	0.951	0.050
	Control/WVB/male/Female	F (1.23)=3.161	0.088	0.399
**SOR Active Time**	Control/Vibration	F (1.23)= 2.708	0.113	0.351
	Male/Female	F (1.23)= 0.153	0.689	0.066
	Control/WVB/male/Female	F (1.23)= 4.947	0.036	0.567

**SOR Passive Time**	Control/Vibration	F (1.23)= 2.249	0.147	0.300
	Male/Female	F (1.23)= 0.010	0.917	0.051
	Control/WVB/male/Female	F (1.23)= 2.466	0.129	0.324

Table 3. T-test to 50% of chance level

	**pWBV♂**	**pWBV♀**	**WBV♂**	**WBV ♀**
NO-Pref vs. 50%	p=0.0533	p=0.0084	p=0.1026	p=0.0213
NL-Pref vs. 50%	p=0.6158	p=0.5361	p=0.0075	p=0.0388

Table 4. Correlation

**Variable**	**OF Wall Time**	**OF Non Wall Time**	**OF Wall Bouts**	**OF NonWall Bouts**	**OF Crossing**	**OF Rearing Time**	**NO Pref.**	**NO Novel Bouts**	**NO Familiar Bouts**	**NL Pref.**	**NL Novel Bouts**	**NL Familiar Bouts**
**Grip**	-.4083	.4083	.0081	.1546	-.0440	.1650	-.1774	-.3001	.0125	.2528	.3771	-.2012
	p=.034	p=.034	p=.968	p=.441	p=.828	p=.411	p=.454	p=.199	p=.958	p=.282	p=.101	p=.395
**Balance Beam**	.3287	-.3287	-.1757	-.3307	-.2667	-.0209	-.0595	.0212	.0455	-.1860	-.1574	.0440
	P=.094	P=.094	P=.381	P=.092	p=.179	p=.918	p=.803	p=.929	p=.849	p=.432	p=.508	p=854
